# Neonatal lupus erythematosus successfully treated by exchange transfusion: a case report and literature review

**DOI:** 10.3389/fped.2024.1470323

**Published:** 2024-10-25

**Authors:** Minqian Zhou, Wenqiang Sun, Hanghang Peng, Xueping Zhu

**Affiliations:** Department of Neonatology, Children’s Hospital of Soochow University, Suzhou, China

**Keywords:** neonatal lupus erythematosus, pancytopenia, congenital heart block, exchange transfusion, case report

## Abstract

**Introduction:**

There are few reports of severe hematological involvement in children with neonatal lupus erythematosus (NLE) treated with exchange transfusion. In this case report, we present a female patient with NLE admitted to the Children's Hospital of Soochow University. The main clinical manifestations were pancytopenia and congenital heart block (CHB). Her condition was serious and could not be improved by conventional treatment; however, she responded well to exchange transfusion therapy.

**Case presentation:**

A female infant, aged 1 month and 3 days, was admitted to the Children's Hospital of Soochow University owing to the “discovery of thrombocytopenia over 1 month.” She tested positive for anti-SSA IgG, anti-Ro-52 IgG, and anti-mitochondrial M2 antibodies. In contrast, her mother tested positive for ANA (1:320) and anti-Ro/SSA antibodies. The patient was diagnosed with NLE and presented with pancytopenia and CHB. Her cardiac function was normal and no intervention was performed; however, her hematological involvement was more severe, without significant improvement after steroid, intravenous immunoglobulin, and transfusion treatments. After exchange transfusion therapy, the patient significantly improved, and the short-term follow-up prognosis was good.

**Conclusion:**

For patients with NLE presenting with hematological involvement that cannot be improved by conventional treatment or whose condition is serious, exchange transfusion therapy should be considered to reduce antibody titers and improve their condition.

## 1 Introduction

Neonatal lupus erythematosus (NLE) is a rare maternal-acquired disease associated with autoimmune antibodies and has an incidence of approximately 1:20,000 (1, 2). It is mainly characterized by skin damage and cardiac, hematological, digestive, and nervous involvement. Antibody titers in most children decrease over time; however, the clinical manifestations improve accordingly, with generally good prognoses except in congenital heart block (CHB) (1, 3). This article reports on a patient with NLE who was admitted to the Children's Hospital of Soochow University in January 2024. The main clinical manifestations were severe hematological involvement and CHB. Her symptoms did not improve after conventional treatment; however, after the exchange transfusion, the short-term prognosis became good. Therefore, we shared her diagnosis and complete treatment process, with the aim of improving the understanding of this disease and providing diagnostic and treatment ideas.

## 2 Case description

A female infant, aged 1 month and 3 days, was admitted to the Children's Hospital of Soochow University on 14 January 2024, owing to thrombocytopenia persisting for over 1 month. The child, the firstborn, was delivered through cesarean section, weighing 2,050 g, with meconium-stained amniotic fluid and an Apgar score of 6–8 points at birth.

After birth, the patient was hospitalized at the birth hospital from 11 to 20 December 2023, during which the thrombocytopenia was first discovered. The lowest platelet count dropped to 16 ×10^9^/L, hemoglobin to 105 g/L, white blood cell (WBC) count to 1.67 ×10^9^/L, and neutrophil count to 0.5 ×10^9^/L. In addition, the patient exhibited atrial flutter, supraventricular tachycardia, ventricular premature beats, and other arrhythmias. During hospitalization, autoantibody profile tests and whole-exome sequencing were performed; however, the results were not available. Despite treatment, including respiratory support, anti-infection, anti-arrhythmia, and transfusions, no improvement was observed. After discharge, the birth hospital called to indicate that the child tested positive for antinuclear antibodies (ANA), including ANA > 500 AU/ml, anti-SSA IgG > 200 AU/ml, anti-Ro-52 IgG 134.79 AU/ml, and anti-mitochondrial M2 antibodies 34.19 AU/ml. Her mother was advised to undergo comprehensive ANA and rheumatoid factor tests. The results showed she was positive for ANA (1:320), anti-Ro/SSA antibodies, and rheumatoid factor (52.8 IU/ml). The rheumatoid factor was two to three times higher than the normal value. Despite this, the mother's medical history revealed no symptoms suggestive of systemic lupus erythematosus or Sjögren's syndrome, such as skin lesions, arthritis, dry eyes, dry mouth, serositis, or cardiac, hematological, or lung involvement. As a result, treatment was not initiated for the patient’s mother.

The patient was hospitalized intermittently at a local children's hospital (23–24 December 2023 and 29 December–14 January 2023). Bone marrow smears indicated a reduced granulocyte proportion, slightly increased eosinophils, 10 megakaryocytes, 2 naked nuclei, and scarce platelets, excluding bone marrow hematopoietic abnormalities. During this period, the patient received three platelet transfusions, one suspension of leukocyte-poor red blood cells (RBCs), three doses of intravenous immunoglobulin (IVIG) (1 g/kg), prednisone tablets (1 mg/kg q12h; 2–9 January 2024), methylprednisolone (MP), sodium succinate (1 mg/kg q12h; 10–14 January 2024), and antibiotics using cefoperazone and sulbactam sodium. The platelet count was in the range of 7–235 × 10^9^/L. After a platelet transfusion on 12 January, the platelet count was 235 × 10^9^/L, prompting hospitalization at our hospital on 14 January 2024.

**Figure 1:**
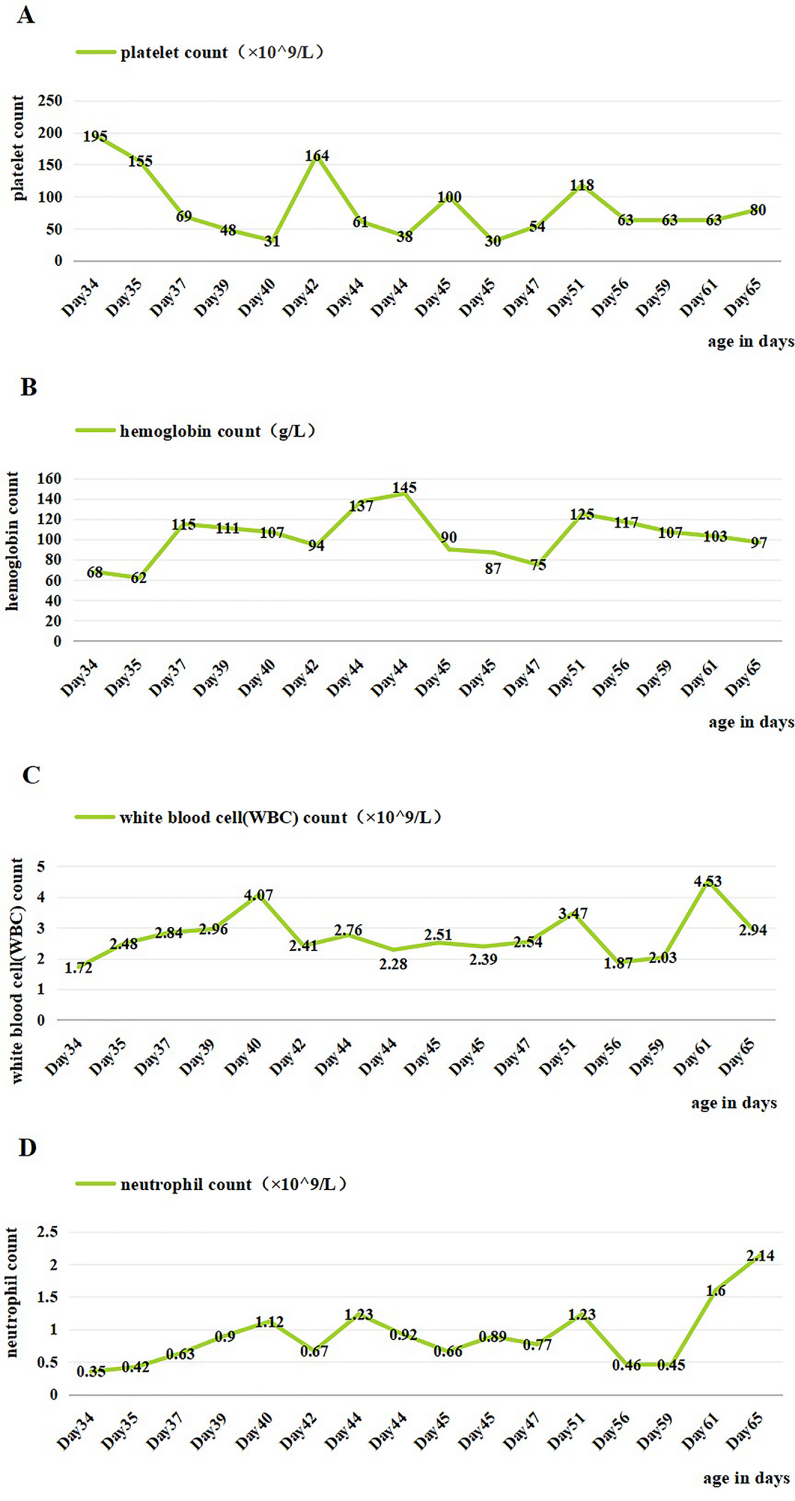
Changes of blood cells in the patient during hospitalization. **(A)** Changes in platelet count during hospitalization. **(B)** Changes in hemoglobin count during hospitalization. **(C)** Changes in white blood cell count during hospitalization. **(D)** Changes in neutrophil count during hospitalization. Day 36, IVIG; days 40 and 46, platelet transfusion; days 35, 36, 48, and 49, suspension of leukocyte-poor red blood cells; day 58, granulocyte-stimulating factor.

On admission, physical examination showed a temperature of 37.4°C, pulse rate of 140 beats/min, respiratory rate of 40 breaths/min, blood pressure of 75/48 mmHg, weight of 2,250 g, and SpO_2_ at 98%. The patient was alert with pale skin, thin subcutaneous fat, warm extremities, regular heart sounds and rhythm, and a grade 2/6 cardiac murmur. She was given box-type oxygen uptake, high-calorie formula feeding, and regular monitoring of blood counts, including platelets, hemoglobin, WBCs, and neutrophils (Figure 1). The reticulocyte percentage and the absolute count were 4.87% and 0.1052 × 10^12^/L, respectively. The pancytopenia led to the administering of preventive antibiotics using ceftriaxone and platelet-raising therapy with MP (2.5 mg q12h). The patient’s blood type was identified as RH-positive A type.

**Figure 2:**
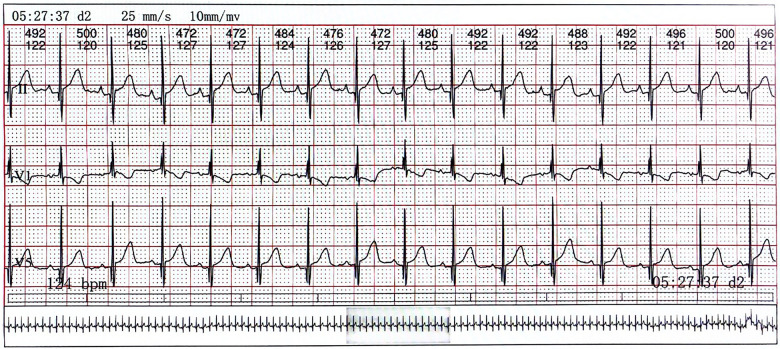
The electrocardiograph of the child. Lead V1 exhibits QRS group “M” type with low ST segment and inverted T wave, indicating right bundle branch block.

On day 2 of admission, mild inspiratory retraction signs were visible, and the platelet and hemoglobin counts dropped to 155 ×10^9^/L and 62 g/L, respectively, prompting the transfusion of a 35 ml suspension of leukocyte-poor RBCs. Considering the low immunity, ceftriaxone was replaced with cefoperazone and sulbactam sodium. On day 3, another 35 ml RBC transfusion was administered, with MP increased to 4.5 mg q12h and IVIG for platelet elevation, alongside fluconazole (FCZ) for fungal infection prevention. On day 4, a 24-h dynamic electrocardiograph (ECG) was conducted and showed sinus arrhythmias, occasional atrial premature beats, and an incomplete right bundle branch block (Figure 2). A cardiology consultation suggested continuation of the current treatment and follow-up for review. On day 5, a consultation with the immunology department confirmed the diagnosis of NLE, and an exchange transfusion was recommended.

On days 6–10, an exchange transfusion was not actively performed, and the patient developed hemorrhagic spots on the chest and abdomen. There was a progressive decrease in platelets down to 31 × 10^9^/L, and the other blood cell counts, with positive ANA and strongly positive anti-SSA antibodies. Conventional treatments proved ineffective as they only inhibited the production of new immune complexes. However, the child had high titers of autoantibodies, leading to the continuous production of new immune complexes. Exchange transfusion can rapidly remove circulating autoantibodies and immune complexes. Finally, on day 11 of admission, the child underwent an exchange transfusion after ineffective treatment with steroids, IVIG, and transfusion. During the procedure, 300 ml of A-type RH-positive washed RBCs was transfused into the right femoral vein, 100 ml AB-type normal frozen plasma was transfused into the superficial temporal vein, and 400 ml of blood was withdrawn from the left axillary artery. It proceeded smoothly.

Genetic testing from an external hospital revealed no lupus-related gene mutations. A clear diagnosis of NLE was made based on the patient's clinical symptoms, positive maternal antibodies, and genetic testing results. Five days after the exchange transfusion, the rechecked ANA profile showed positive anti-SSA antibodies, weakly positive anti-Ro-52 antibodies, and questionably positive anti-AMA-M2 antibodies, indicating improvement. However, the elevation of platelets, hemoglobin, WBCs, and neutrophil counts were not obvious. As a result, the infant continued to receive transfusion, vitamin K1, granulocyte-stimulating factor (GSF), antibiotics, MP, and FCZ. Hematological involvement improved significantly, with stable blood cell counts. The patient was discharged on 15 February 2024.

**Figure 3:**
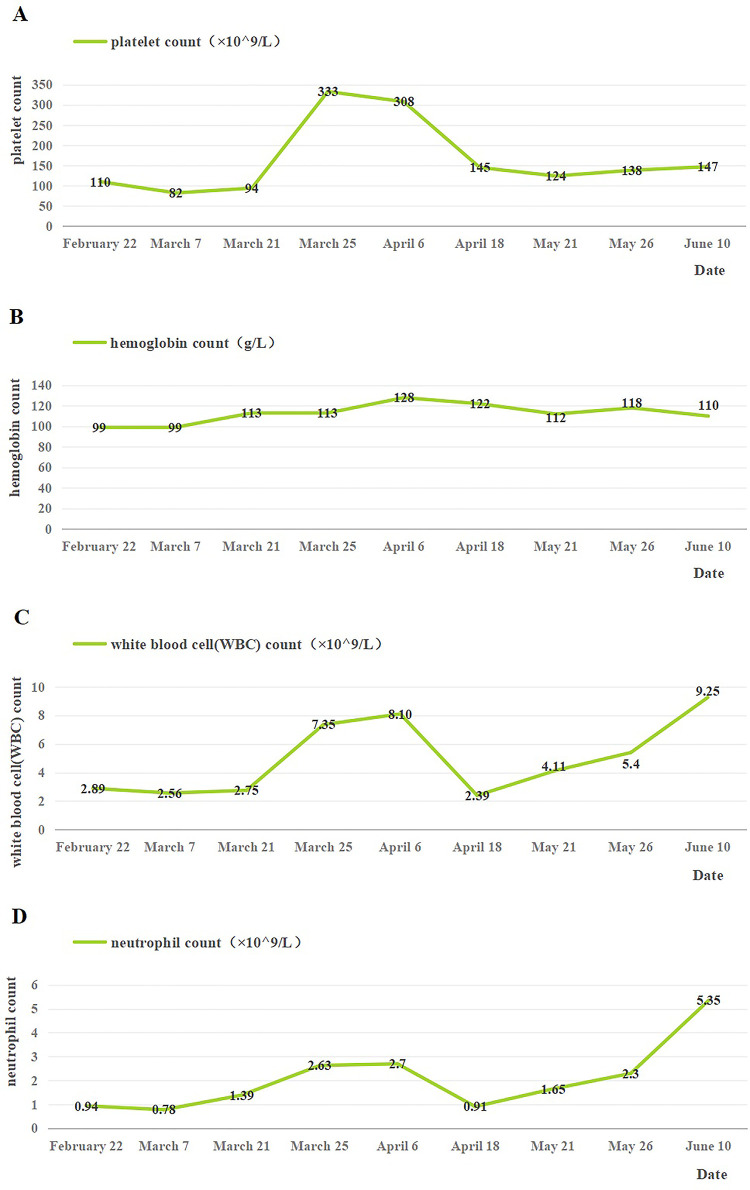
Changes of blood cells in the patient after discharge. **(A)** Changes in platelet count after discharge. **(B)** Changes in hemoglobin count after discharge. **(C)** Changes in white blood cell count after discharge. **(D)** Changes in neutrophil count after discharge. 18 April: the use of MP was stopped.

The patient was continued on MP (15 February to 18 April) and burned root leukopoietic cells (22 February to late May). Seven months after discharge, the patient underwent nine routine blood investigations. The platelet count was in the range of 82–333 ×10^9^/L, the hemoglobin level was maintained at 99–128 g/L, the WBC count was in the range of 2.39–9.25 ×10^9^/L, and the neutrophil count fluctuated between 0.78 and 5.35 ×10^9^/L (Figure 3). After discharge, blood cell levels increased; however, after the withdrawal of MP on 18 April, there was a temporary decline and a steady increase. However, ANA was re-investigated 4 months after discharge and the results were negative. In general, the short-term follow-up prognosis was good. Electrocardiograms have not yet been reviewed, and long-term follow-up results are still needed.

## 3 Discussion

NLE is a maternally acquired disease associated with autoimmune antibodies, such as anti-Ro/SSA antibodies (anti-Sjögren's syndrome A antibodies, also known as anti-Ro antibodies), anti-La/SSB antibodies (anti-Sjögren's syndrome B antibodies, also known as anti-La antibodies), and, less commonly, anti-U1-ribosomal protein antibodies (1). The incidence of NLE is approximately 1:20,000 (2). The currently recognized diagnostic criteria for NLE include lupus-associated skin damage or CHB, with either neonatal and maternal anti-Ro/SSA antibodies and/or anti-La/SSB antibodies (1, 4). The patient's symptoms and test results were consistent with the diagnostic criteria. Genetic testing did not reveal any primary lupus-related genetic markers, thereby confirming the diagnosis.

The clinical manifestations of NLE often include skin damage as well as involvement of the cardiac, hematological, digestive, and nervous systems (1, 3). Cardiac involvement can include electrophysiological abnormalities, cardiomyopathy, myocarditis, or structural cardiac abnormalities, of which the most severe is CHB (5). Erden et al. (6) showed that the incidence of CHB in patients with NLE in Europe and America was 49.4% and 35.0%, respectively. In contrast, the incidence in Asia was only 11.4%. The incidence of skin damage in Asian patients was 45.2%, which was significantly higher than that in European and American patients (16.8% and 37.6%, respectively). Li et al. (7) discovered that in 123 Chinese patients with NLE, skin damage was present in approximately 96.0% of cases, whereas cardiac involvement was present in only 8.9% of cases. Studies suggest that anti-SSA antibodies are associated with CHB, whereas anti-SSB antibodies are associated with generalized skin damage (8, 9). This may explain the presence of CHB without skin damage in this patient.

NLE has a favorable prognosis; however, the mortality rate increases when accompanied by CHB, mainly due to fatal complications such as dilated cardiomyopathy, heart failure, and endocardial fibroelastosis (5, 10). After progression to complete CHB, 60% of patients require a cardiac pacemaker (8, 11). In this case, the child presented with incomplete CHB without adverse complications; therefore, no special treatment was necessary, and dynamic observation was recommended. The patient also exhibited severe hematological involvement. Song discovered that 51.9% of patients with NLE had hematological involvement, including anemia (18.5%), thrombocytopenia (11.1%), and granulocytopenia (11.1%); some children had combined symptoms (3.7%) (12). Li et al. retrospectively analyzed 123 children with NLE in China and found that 56 presented with hematological abnormalities, of which 17 (13.82%) were thrombocytopenic, 38 (30.89%) were anemic, and 12 (9.75%) were thrombocytopenic and anemic (7). In patients with NLE, hematological involvement is usually minimal; laboratory test results show mild decreases and no relevant clinical symptoms that may recover within 1 year (13, 14). However, this patient had thrombocytopenia, anemia, leukopenia, and granulocytopenia, indicating significant involvement of the blood system. The patient's pancytopenia did not significantly improve with steroids, IVIG, or transfusion, likely due to the high titers of antibodies. The clinical manifestations significantly improved after the exchange transfusion, with no worsening observed during short-term follow-up.

Currently, there is no consensus regarding treatment for NLE. Relevant studies have shown that hydroxychloroquine (HCQ), a toll-like receptor inhibitor, can be used to prevent NLE cardiac involvement during pregnancy; however, there are no reports of its off-label use in newborns (15). Steroids prevent the progression of incomplete CHB to complete CHB caused by NLE (5), improve skin damage, and reduce the formation of new immune complexes (16). The effectiveness of IVIG in treating NLE depends on the Fc receptors (17). In 80% of patients, IVIG improves platelet counts within 1–4 days; however, this effect lasts for only 1–2 weeks. In addition, the combined use of steroids may be more effective (18). Children with thrombocytopenia and anemia may require blood component transfusion. In addition to drug treatment, blood purification therapy can rapidly eliminate antibodies from the body and prevent further damage. Children with NLE often have low blood volume. Plasma exchange can treat autoimmune diseases by removing immune complexes, IgM, and other substances, and it can rapidly reduce IgG levels to effectively alleviate critical complications, such as thrombocytopenia, in the acute phase (19). Reports indicate that exchange transfusion therapy is an effective treatment for NLE, offering a higher antibody clearance rate and significant efficacy (20). We used a 3:1 ratio of washed RBCs to ordinary frozen plasma for the patients’ transfusion, which reduced the risk of albumin loss and was safer. However, as the elimination of antibodies mainly depends on plasma, increasing the proportion of plasma under security may be considered. If the effect is not satisfactory, the blood exchange is repeated. The child was considered to have low immunity because of her young age and prematurity. Owing to the presence of infection, long-term antibiotics were administered; however, her temperature remained within the normal range, she was alert, and her blood culture was negative. Therefore, there is controversy regarding the selection and duration of antibiotic use. The patient presented with hematological involvement and CHB, with a normal cardiac function despite incomplete CHB. Therefore, no interventional measures were taken. The hematological involvement was severe, and there was no significant improvement after conventional treatment with steroids, IVIG, and transfusion. Her antibody titers were markedly elevated, which were significantly improved by exchange transfusion therapy. Her parents expressed gratitude for the diagnosis, treatment, and good prognosis their child received at the hospital.

For patients with NLE presenting with hematological involvement that does not respond to conventional treatment or those in a serious condition, exchange transfusion therapy should be considered to reduce antibody titers and improve their overall condition.

## Data Availability

The original contributions presented in the study are included in the article/Supplementary Material, further inquiries can be directed to the corresponding author.
